# MIDRC-MetricTree: a decision tree-based tool for recommending performance metrics in artificial intelligence-assisted medical image analysis

**DOI:** 10.1117/1.JMI.11.2.024504

**Published:** 2024-04-03

**Authors:** Karen Drukker, Berkman Sahiner, Tingting Hu, Grace Hyun Kim, Heather M. Whitney, Natalie Baughan, Kyle J. Myers, Maryellen L. Giger, Michael McNitt-Gray

**Affiliations:** a University of Chicago, Department of Radiology, Chicago, Illinois, United States; b U.S. Food and Drug Administration, Bethesda, Maryland, United States; c University of California Los Angeles, Los Angeles, California, United States; d Puente Solutions, Phoenix, Arizona, United States

**Keywords:** artificial intelligence, machine learning, computer-aided diagnosis, performance evaluation

## Abstract

**Purpose:**

The Medical Imaging and Data Resource Center (MIDRC) was created to facilitate medical imaging machine learning (ML) research for tasks including early detection, diagnosis, prognosis, and assessment of treatment response related to the coronavirus disease 2019 pandemic and beyond. The purpose of this work was to create a publicly available metrology resource to assist researchers in evaluating the performance of their medical image analysis ML algorithms.

**Approach:**

An interactive decision tree, called MIDRC-MetricTree, has been developed, organized by the type of task that the ML algorithm was trained to perform. The criteria for this decision tree were that (1) users can select information such as the type of task, the nature of the reference standard, and the type of the algorithm output and (2) based on the user input, recommendations are provided regarding appropriate performance evaluation approaches and metrics, including literature references and, when possible, links to publicly available software/code as well as short tutorial videos.

**Results:**

Five types of tasks were identified for the decision tree: (a) classification, (b) detection/localization, (c) segmentation, (d) time-to-event (TTE) analysis, and (e) estimation. As an example, the classification branch of the decision tree includes two-class (binary) and multiclass classification tasks and provides suggestions for methods, metrics, software/code recommendations, and literature references for situations where the algorithm produces either binary or non-binary (e.g., continuous) output and for reference standards with negligible or non-negligible variability and unreliability.

**Conclusions:**

The publicly available decision tree is a resource to assist researchers in conducting task-specific performance evaluations, including classification, detection/localization, segmentation, TTE, and estimation tasks.

## 1 Introduction

The Medical Imaging and Data Resource Center (MIDRC) was established in response to the coronavirus disease 2019 (COVID-19) pandemic.^1^ It is a collaborative initiative funded by the National Institute of Biomedical Imaging and Bioengineering and hosted at the University of Chicago. MIDRC is co-led by the American College of Radiology® (ACR®), the Radiological Society of North America (RSNA), and the American Association of Physicists in Medicine (AAPM). Its primary objective is to facilitate machine learning (ML) innovation through data sharing, enabling rapid and flexible collection, analysis, and dissemination of imaging and associated clinical data. By providing researchers with extensive resources, MIDRC aims to support their efforts in combating COVID-19. Note that while many of these resources were developed for the medical image analysis use case of COVID-19, they can be extended to other diseases, i.e., other use cases. Specifically, MIDRC focuses on three key areas: (1) creating a large, publicly available resource of curated medical images and associated metadata, which can be utilized to develop artificial intelligence (AI)/ML algorithms addressing clinical tasks such as detection, diagnosis, prognosis, and treatment response assessment, (2) establishing a sequestered dataset that is not accessible to the public, designed to facilitate unbiased algorithm assessment and regulatory approval processes, and (3) developing resources and tools to assist medical imaging AI/ML researchers in their work.

This work falls under the third key area mentioned above and aims to provide a user-friendly interactive decision tool, the MIDRC-MetricTree,^2^ to help researchers and clinicians make informed decisions about how to evaluate the performance and reliability of a medical image analysis AI/ML model with appropriate metrics. It is worth noting that finding a single “ideal” performance metric for evaluating AI/ML in medical imaging tasks is challenging, and it is common to use multiple evaluation metrics to provide a more comprehensive evaluation of performance. Different metrics capture different aspects of performance, and each metric provides a unique perspective and addresses specific considerations. For example, sensitivity and specificity are relevant for diagnostic accuracy, precision and recall curves are important for evaluating the trade-off between false negatives and positive predictive value, and the area under the receiver operating characteristic (ROC) curve provides an overall measure of discrimination. Therefore, in this example, these three complementary sets of metrics provide a more comprehensive evaluation of system performance than any one metric by itself. In addition, it is important to consider the clinical context and specific task requirements when selecting and interpreting performance metrics. For example, special considerations need to be made in cases of class imbalance. The desired or required performance levels also depend on the setting (research versus clinical practice) and application (e.g., organ segmentation versus lesion segmentation). It should also be noted that reporting uncertainty estimates, such as 95% confidence intervals, is crucial for understanding the reliability and generalizability of AI/ML models in medical imaging tasks since confidence intervals provide a range of values within which the true performance of the model is likely to fall, indicating the uncertainty associated with the estimated performance metrics. Many of the issues related to selecting approaches, selecting evaluation metrics, handling unusual scenarios, and reporting performance are addressed in the tree, and in many cases, reference material is provided.

AI/ML algorithms have been proposed for a wide variety of clinical medical image analysis tasks currently performed by radiologists, including diagnosing diseases, distinguishing among different disease types, localizing abnormalities, determining disease extent, estimating severity and physiological parameters, and predicting patient prognosis. These algorithms have the potential to assist radiologists in performing these tasks by improving interpretation accuracy, efficiency, and patient outcomes by automating certain aspects of clinical tasks. To be useful and ultimately adopted into clinical practice, the performance of AI/ML algorithms needs to be rigorously evaluated. In this work, by emphasizing the importance of appropriate performance evaluation metrics, we aim to foster the development of AI/ML algorithms that meet the rigorous standards required for successful integration into the clinical practice of the future. Though our work was initially motivated by the COVID-19 pandemic and our efforts were focused on addressing this disease, we have generalized our approach to allow these efforts to be applicable to other radiological tasks, i.e., other medical image analyses for which AI/ML algorithms might be developed.

In this paper, we present our suggested categories of tasks and performance metrics, which have been incorporated into our decision tree tool. We provide guidance on how to navigate the evaluation process effectively and give a high-level overview of the decision tree. For each section of the tree (i.e., for each task), we have provided an overview of the task, a simplified flowchart of the decision tree (indicating branches where the user chooses a direction based on details of their algorithm, including the type of data used and the reference standard being used for evaluation), and relevant references where appropriate.

## 2 Methods

Our first step was to identify the various roles that imaging might play in the detection, diagnosis, assessment of treatment response, and prognosis of COVID-19. Then, we expanded to consider other diseases in which radiology and medical image analysis play a role. We also considered that the imaging might be performed using different imaging modalities (radiography, CT, MR, PET-CT, etc.) and that the imaging might be performed at different times (before and after diagnosis of disease). To capture the nuances and challenges associated with each of the many radiological tasks and imaging modalities, task-specific performance metrics must be thoughtfully selected. To this end, we conducted an extensive literature review and engaged in discussions with a mathematical statistician and a medical regulatory science expert to identify generalized categories of tasks, i.e., types of tasks, relevant to radiologists and corresponding performance metrics. We then synthesized our findings into an interactive decision tree tool, to serve as an extensive and practical guide for AI/ML developers to evaluate the performance of their algorithms across various stages of development, from inception to potential clinical deployment. By utilizing this tool, developers can step through the evaluation process, consider several complementary performance metrics, and gain insight into the reliability and utility of their medical image analysis AI/ML algorithms.

In the development of our interactive decision tree tool, the first step was to identify best practices and recommended metrics for each of the different medical image analysis AL/ML tasks that we had identified, considering that different AI/ML output types and reference standards (“ground truth”) may be used for each task. We identified appropriate, preferably open-source, software/code, and tutorial videos to assist users of the tree to identify and understand the approaches being recommended. We worked to design our decision tree to provide, whenever possible, multiple appropriate metrics, ways to calculate error estimates, and special considerations while keeping the structure of the tree “branches” and “nodes” as consistent as possible for the different identified AI/ML tasks.

## 3 Results

**Fig. 1:**
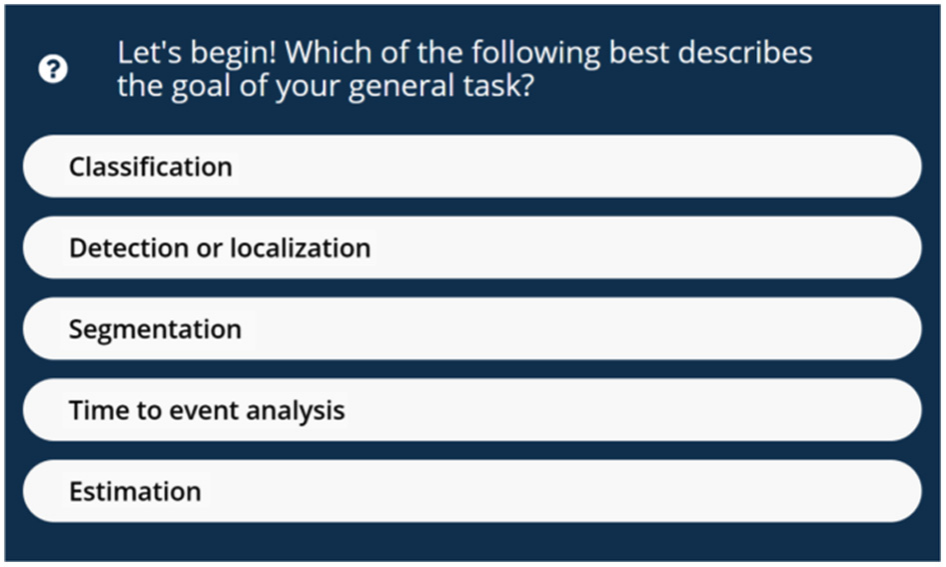
User interface of the start node of the MIDRC-MetricTree.

**Fig. 2:**
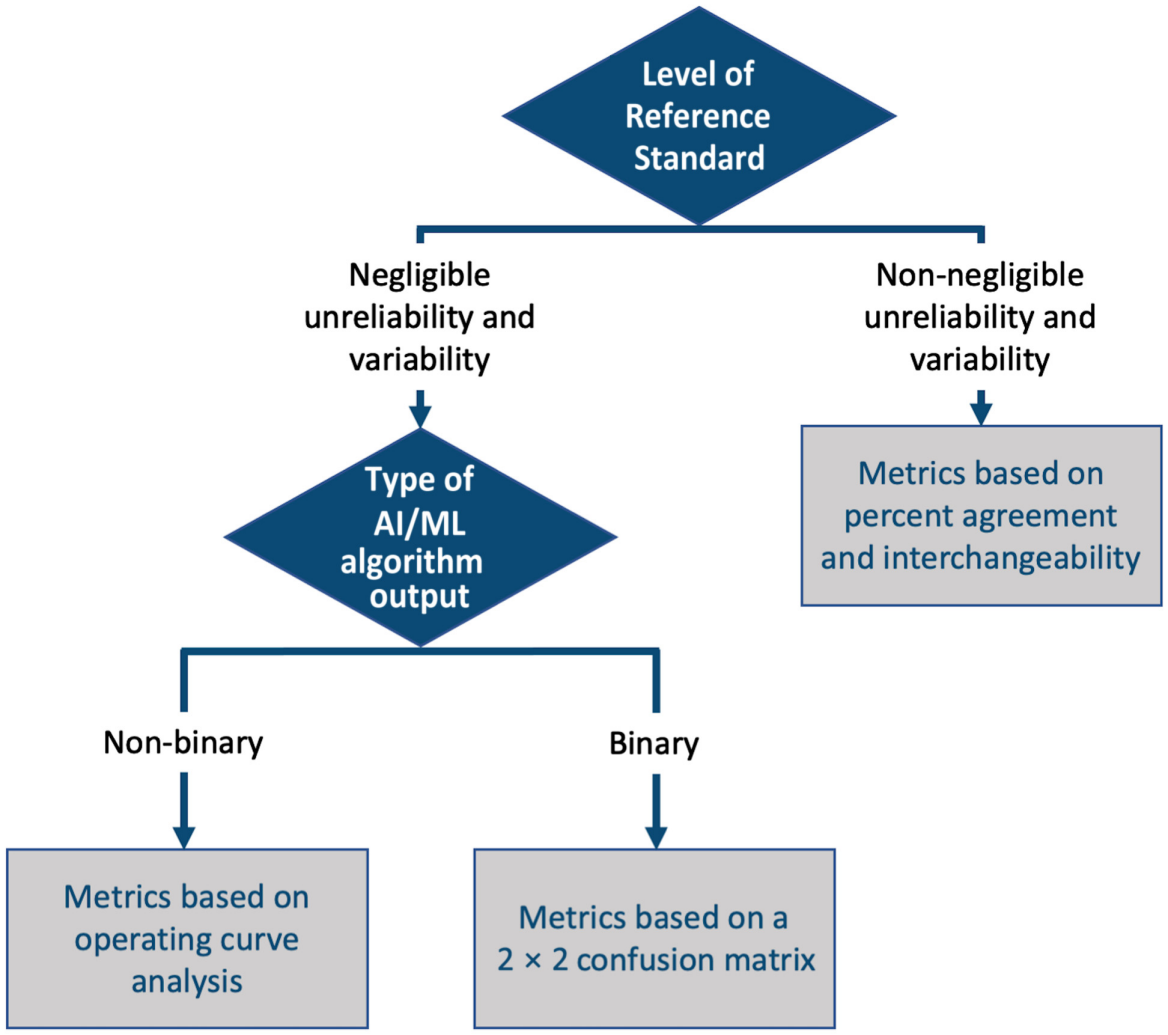
Simplified flowchart of the binary classification branch of the MIDRC-MetricTree.

We identified five types of clinical medical image analysis tasks of interest (Fig. 1): (1) classification, (2) detection or localization, (3) segmentation, (4) time-to-event (TTE) analysis, and (5) estimation. We organized our interactive decision tree tool, the MIDRC-MetricTree^2^ hosted on the MIDRC website, by the type of the identified tasks. In this decision tree, a user can select information, such as the type of task, the nature of the reference standard, and the type of algorithm output. Based on the responses provided, a user then obtains recommendations regarding appropriate performance evaluation approaches and metrics, as well as literature references, short video tutorials, glossaries, and links to available software and/or code when applicable. The MIDRC-MetricTree may suggest multiple metrics, uncertainty estimates, and special considerations based on a user’s input. To help users navigate the decision tree, flowcharts for each task are provided at the starting node for each task (Figs. 2 and 4–7), and “hover text” explains the concepts listed as choices [such as for the type of task (Fig. 1)].

### 3.1 Classification Tasks

**Fig. 3:**
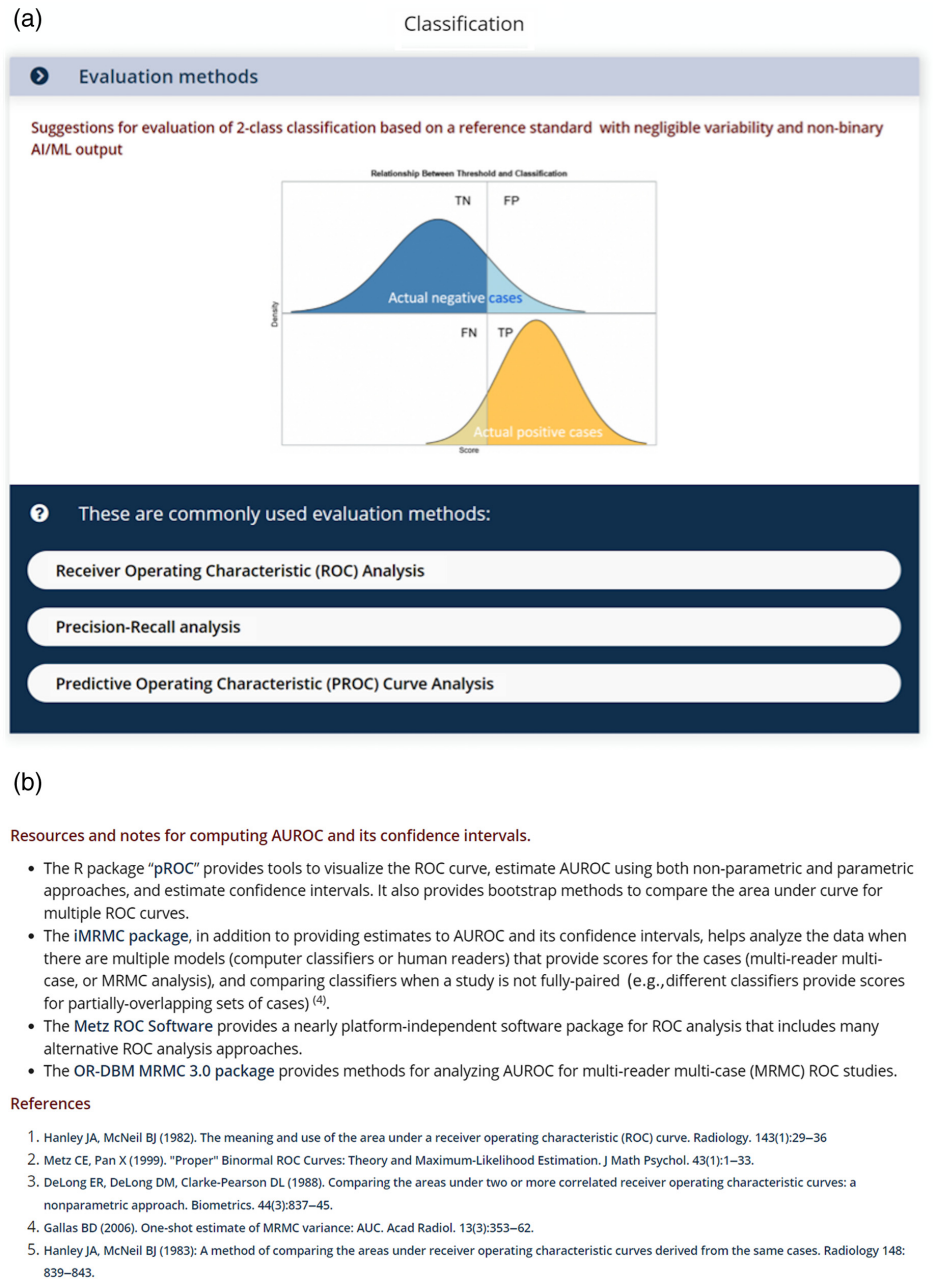
Example of (a) suggested evaluation methods for binary classification with continuous AI/ML output and (b) end node with resulting metrics and additional information. Note that, in this end node, the suggested code/packages and references have links to their sources, and as such, the user can click on these and obtain code/packages and cited literature.

**Fig. 4:**
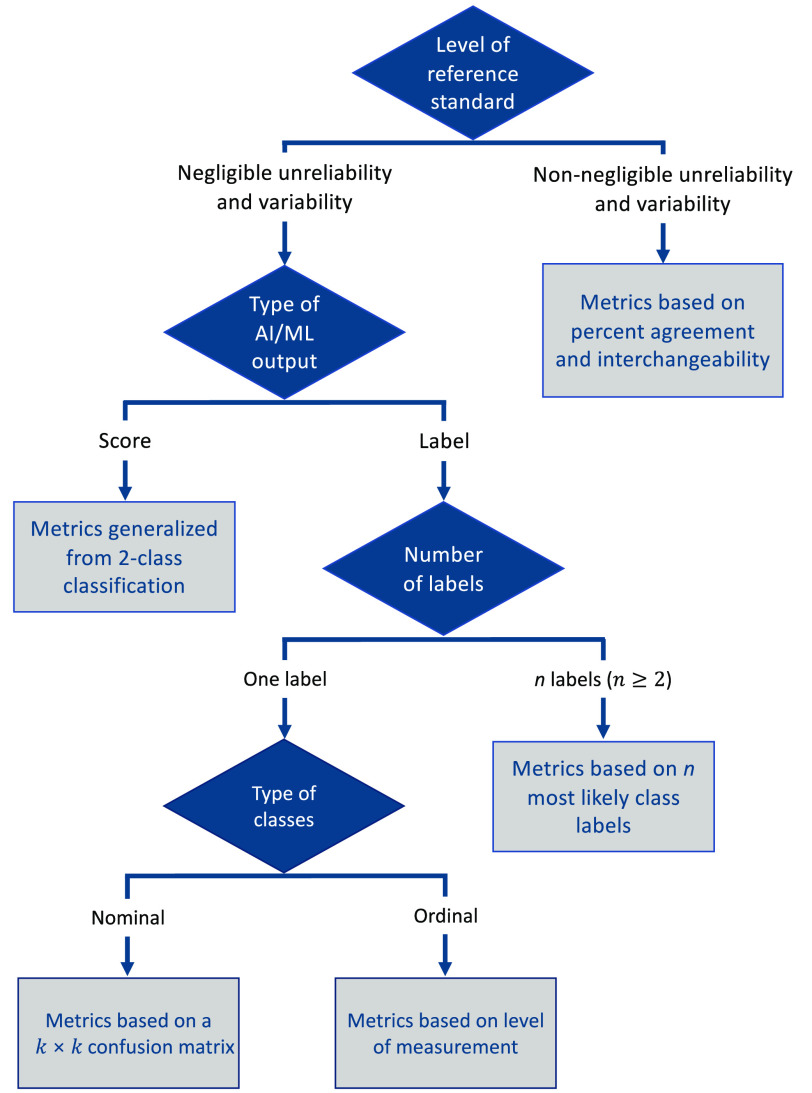
Simplified flowchart of the multiclass classification branch of the MIDRC-MetricTree.

The classification branch of the decision tree was developed for binary (two-class, Figs. 2 and 3) and multiclass (more than two classes, Fig. 4) classification tasks. An example of a binary classification task is the classification of COVID-19-positive versus COVID-19-negative chest radiographs. An example of a multiclass (N=3 classes) classification task is the distinction among chest radiographs depicting either community-acquired pneumonia, COVID-19-associated pneumonia, or normal lungs.

For the binary classification task (Fig. 2), users are asked about the reliability and variability in their reference standard and the type of their ML output, which can be binary (e.g., yes/no presence of disease) or non-binary (e.g., continuous as in a probability of disease). The user’s responses to these two questions lead to one of the following three possibilities:

1. binary classification based on a reference standard with negligible variability and binary ML output
2. binary classification based on a reference standard with negligible variability and non-binary ML output
3. binary classification based on a reference standard with non-negligible variability.

Note that in this context, reliability and variability pertain to the likelihood that the labels/annotations for all cases are reproducible. The assessment of this level of reliability and variability is somewhat subjective: “negligible unreliability or variability” means that there is high certainty that the “truthing” process would yield the same labels/annotations for all cases when repeated, e.g., because it relies on a consistent laboratory test result such as COVID-19 positive versus COVID-19 negative. On the other hand, “non-negligible unreliability or variability” means that there is substantial deterministic or random error in the reference standard, e.g., because it relies on human assessment, such as visual assessment of severe versus non-severe disease. In the penultimate nodes of the binary classification branch, the user is shown a list of recommended evaluation methods, e.g., ROC analysis^3^ [Fig. 3(a)], and in the ultimate nodes, metrics associated with the evaluation method of choice are provided, e.g., area under the curve from ROC analysis [Fig. 3(b)].

The structure of the branch for multiclass classification (Fig. 4) is similar to that of the binary classification branch, except that there is an additional consideration for the possibility that the AI/ML output is a label (e.g., “COVID-19-pneumonia,” “non-COVID-19 pneumonia,” or “normal”), rather than a score, and whether this output provides *one* or *multiple* most likely class labels. Subsequently, there are four penultimate nodes describing evaluation methods for scenarios:

1. multiclass classification based on a reference standard with negligible variability and a single-label output
2. multiclass classification based on a reference standard with negligible variability and an output of n (n>1) mostly likely class labels
3. multiclass classification based on a reference standard with negligible variability and output of class scores
4. multiclass classification based on a reference standard with non-negligible variability.

Metrics and additional information are again provided in the ultimate nodes.

### 3.2 Detection or Localization Tasks

Detection or localization plays a crucial role in medical image analysis, such as the computer-aided detection of cancerous lesions in mammograms or the localization of ground-glass opacities associated with COVID-19 pneumonia in chest radiographs. The evaluation of performance in detection or localization tasks heavily relies on the definition of true-positive, false-positive, and false-negative detections.^4^ These definitions categorize detections as “correct” or “incorrect,” and targets as “detected” or “missed.” In several scenarios, different criteria are used to determine true positives, false positives, and false negatives, considering the reference standard and the detection candidate(s). These criteria encompass distances between detections and the reference standard, as well as overlap criteria described in the segmentation branch (Sec. 3.3). Strategies to reduce false positives include the consolidation of detections through similar criteria.

**Fig. 5:**
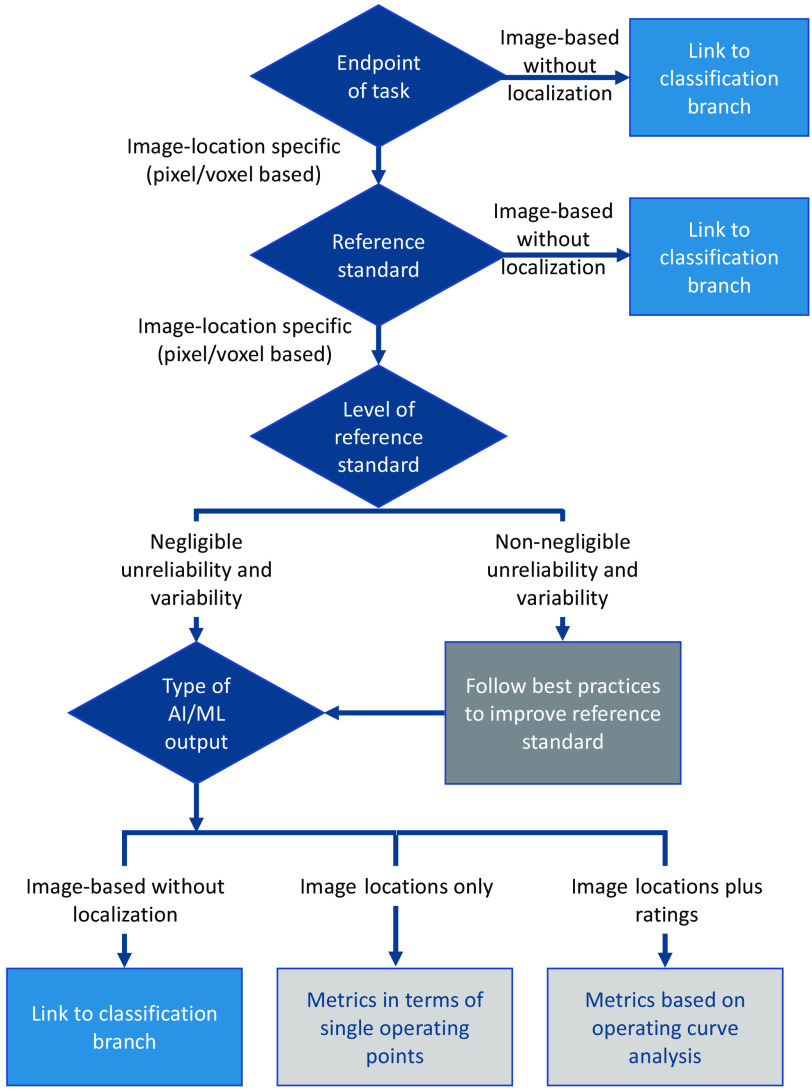
Simplified flowchart of the detection/localization branch of the MIDRC-MetricTree.

In the detection branch, the analysis encompasses several aspects, including the task endpoint, the granularity and variability/reliability of the reference standard, and the type of AI/ML output (Fig. 5). It is noted that when humans determine reference standard bounding boxes, centroids, or manual outlines (as is most often the case), a degree of variability is unavoidable. Best practices involve multiple annotators providing annotations, followed by curation through arbitration or consensus.^5^

Performance evaluation of detection and localization problems often employs precision–recall analysis or free-response operating curves (FROCs).^4^^,^^6^^,^^7^ Recall, also known as sensitivity, reflects the model’s ability to identify *all* relevant objects, while precision denotes the model’s ability to correctly identify *only* relevant objects, measured by the fraction of correct positive predictions (positive predictive value).^4^ Free-response analysis provides sensitivity, representing the total number of correct localizations divided by the total number of objects, along with the total number of incorrect detections divided by the total number of images.^7^

In the detection/localization decision branch, three types of AI/ML outputs are considered:

1. image-based AI/ML output without localization
2. AI/ML output providing image locations/regions only
3. AI/ML output providing image locations/regions along with ratings (e.g., indicating the certainty of a detection representing an actual finding).

For the first scenario, performance assessment is akin to the classification branch since the AI/ML output does not involve localization. In the second scenario, a single operating point, such as a precision–recall or false positives per image and sensitivity operating point, can be employed. In the third scenario, where the AI/ML output includes ratings, precision–recall or FROCs can be generated by utilizing these ratings as decision variables. Recommended performance analysis methods include precision–recall, location ROC, FROC, and alternative FROC (AFROC) analyses.^6^^–^^10^ Derived performance metrics encompass the area under the FROC up to a specific number of false positives per image, area under the AFROC, mean precision, mean average precision, and competitive performance metric.

In the second scenario, it may also be possible to obtain entire operating curves, as in the third scenario, if sufficient information is available on the “raw” detections and the criterion is used to label detections as true or false positives, as well as to identify missed objects. By varying the threshold value for defining a true positive, such as an intersection-over-union threshold, an operating curve can be generated.^4^

### 3.3 Segmentation Tasks

**Fig. 6:**
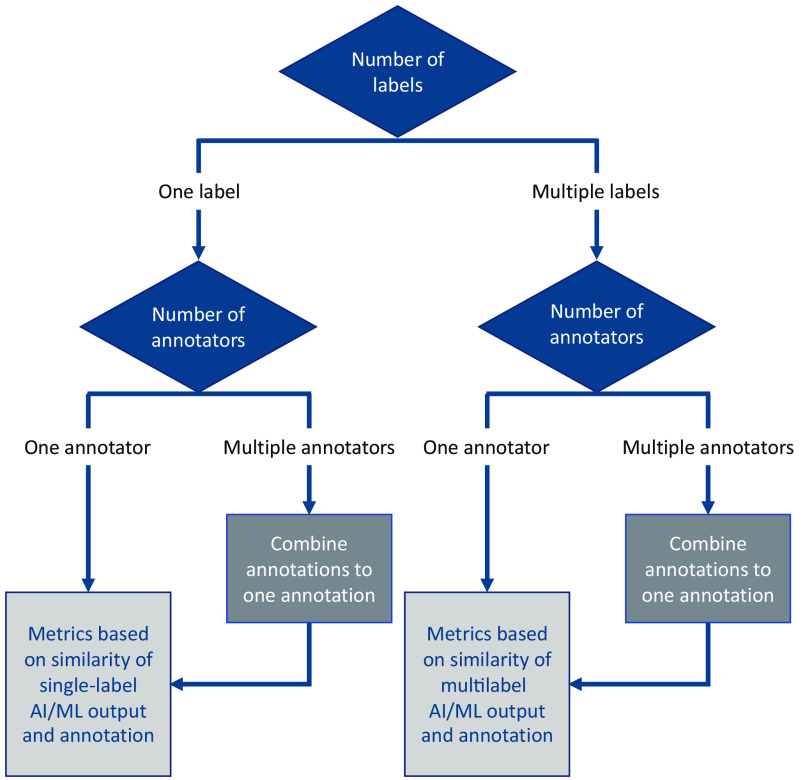
Simplified flowchart of the segmentation branch of the MIDRC-MetricTree.

The segmentation branch of the decision tree describes only methods that compare a computer-generated segmentation to ground truth segmentation(s) generated by one (or multiple) annotator(s) (Fig. 6). Thus, unsupervised segmentation evaluation techniques^11^ that evaluate image segmentation without a reference image are currently out of scope. Two important questions asked to users are (1) the number of different object labels in the AI/ML segmentation algorithm output and (2) the number of annotations involved in defining the ground truth. Most segmentation algorithms in medical imaging are crisp, meaning that each pixel is assigned to only a single object. However, metric definitions for fuzzy classification are easily applied to crisp segmentations, and therefore, the decision tree does not ask the user whether the segmentation is crisp or fuzzy. Whenever a distinction between crisp and fuzzy segmentation is needed, a discussion is provided on the relevant metric description page. Likewise, performance evaluation methods and metrics typically apply in a similar way to two-dimensional (2D) and three-dimensional (3D) segmentations. Therefore, the decision tree does not ask the users whether the imaging modality is 2D or 3D. Note, however, that it is extremely important to use 2D and 3D metrics correctly and that the correlation between 2D image slices of a 3D image volume should not be ignored. Thus, when the metrics described in this branch are used on a dataset to reach statistical conclusions over a population of cases, the correlation among different measurements should be factored into the statistical analysis.

Based on whether there are one or multiple labels (in addition to the background label) in the segmentation task and whether there are one or multiple annotations (serving as truth), there are four possibilities (Fig. 6):

1. one label, one annotator
2. one label, multiple annotators
3. multiple labels, one annotator
4. multiple labels, multiple annotators.

The first scenario (one label, one annotator) forms the major trunk of this branch, as it is the most widely considered/discussed/studied application for medical image segmentation in practice. Several metrics are recommended for this category, which are divided into six groups: overlap-based metrics, volume or area-based metrics, pair counting-based metrics, information-theoretic metrics, probabilistic metrics, and spatial distance-based metrics, following Taha and Hanbury’s work^12^ and the accompanying software.^13^ For the third scenario (multilabel, one annotator), there are only a few suggested metrics due to their complex nature, which are generalizations of the metrics discussed for the first scenario. For the scenarios involving multiple annotators, the most used evaluation method^14^ involves combining annotations from multiple annotators into a consensus annotation, which is then used as the “ground truth” for comparison with the computer-generated segmentations. This approach may not sufficiently account for the variability of the ground truth generated by multiple annotators, however, especially if this variability is high. To our knowledge, at present, no metric or analysis method is commonly agreed upon to adequately consider this variability, which points to a research gap in this area.

### 3.4 TTE Analysis Tasks

**Fig. 7:**
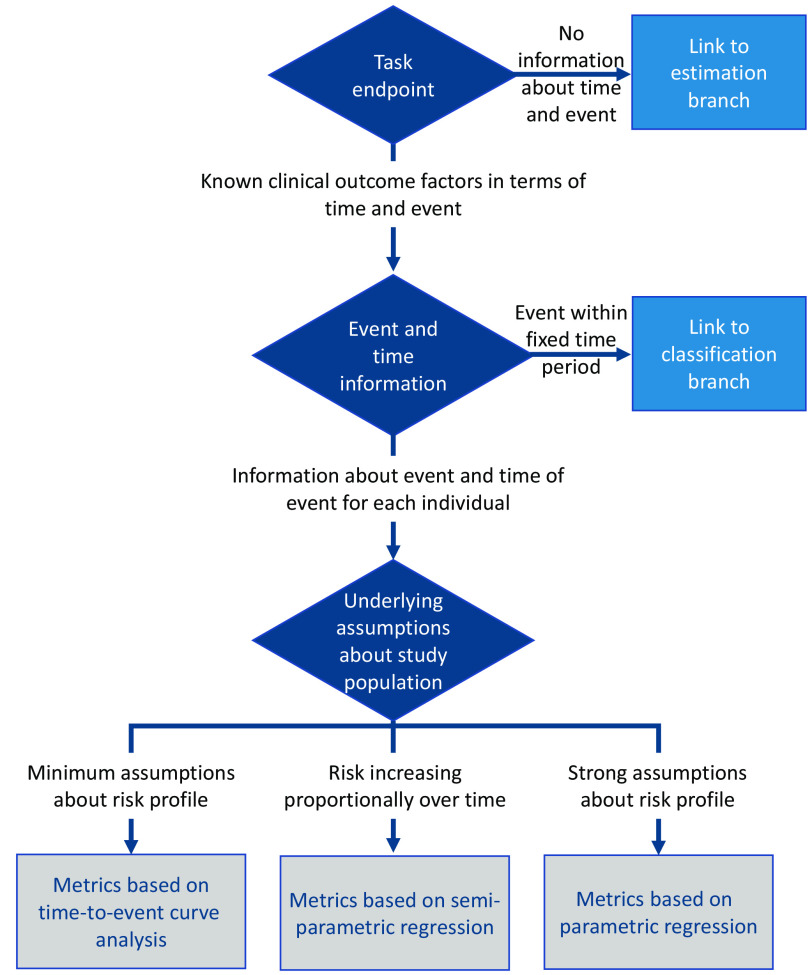
Simplified flowchart of the TTE analysis branch of the MIDRC-MetricTree.

The TTE analysis has two fundamental components—the time variable and the event indicator. The TTE analysis seeks to evaluate not only if an event has occurred but also when it occurred and whether the event occurrence was based on a predictive variable (referred to as a factor). This type of analysis is commonly used in survival analyses or other TTE analyses such as predicting the time to hospitalization or other medical events. The TTE analysis assumes that the following have been defined: (a) the event of interest (e.g., hospitalization and specific treatment intervention), (b) the time (date) that the study period began, and (c) the time (date) that the study period ended. Time can be either the time of the event or the last follow up without the defined event (which is known as “right censoring”). The TTE analysis is based on the process of counting the number of events over time.^15^ If either the temporal or event information is missing, then a TTE analysis is not applicable. For example, if there is an interest in associating a factor or an output from an ML algorithm with some sort of diagnostic assessment, say the algorithm provides an estimate of the severity of COVID-19 from a single time point without a longitudinal comparison and where the reference truth is the visual score (and there is no time or event information available), then the performance evaluation of the method or metric is better suited to an estimation analysis. Another example is if there is information available about events, but it is within a fixed time (e.g., an algorithm that provides a prediction of cancer within a 12-month period), then the performance evaluation is better suited to a classification problem. These alternate pathways are illustrated in Fig. 7.

The TTE analysis is primarily concerned with discovering any potential clinical impact of an intervention or algorithm. The most used test statistics are the logrank test and the Cox regression model.^15^^–^^18^ Although there are several parametric models, such as exponential^19^ and Weibull,^20^ we do not encourage the use of these models unless they are being used for a specific purpose (e.g., pediatric population fitting) or have a strong rationalization due to an understanding that the data will meet the underlying distribution assumptions. The logrank test^15^^,^^16^ is a non-parametric method that is commonly used, and the Cox regression model^17^^,^^18^ is a semi-parametric method with assumptions of a proportional hazard model, for which the assumptions need to be evaluated after model fitting. The contribution of a developed algorithm is performed by testing it as a group variable in the logrank test or as an independent variable, called a hazard ratio (HR), in the Cox regression model. Thus, the recommended approaches are (Fig. 7):

1. Methods based on TTE curve analysis: Kaplan–Meier (KM) plot and logrank test are the most common non-parametric approach in TTE analyses. KM curves can be constructed for TTE endpoints, and the estimate and 95% confidence interval of the median time of the TTE endpoint can be obtained. Logrank test statistics are used where the output of the algorithm is expressed as a categorical variable,^15^^,^^17^ and the null hypothesis is the TTE of categorical groups being equal over time.
2. Methods based on semi-parametric regression: Cox proportional regression model, assuming a proportional hazard over time. A coefficient of HR is tested where the null hypothesis is the HR being equal to 1, and the output of an algorithm is expressed as a categorical or a continuous variable.^17^^,^^21^^–^^24^
3. Methods based on parametric regression: Parametric hazard function, which makes strong assumptions about the underlying risk profile with examples of exponential distribution (Weibull distribution, Gompertz distribution, and log-logistic distribution). However, parametric models are not robust to the misspecification of the underlying distribution of the population, which is why they are not commonly recommended.^25^

### 3.5 Estimation Tasks

**Fig. 8:**
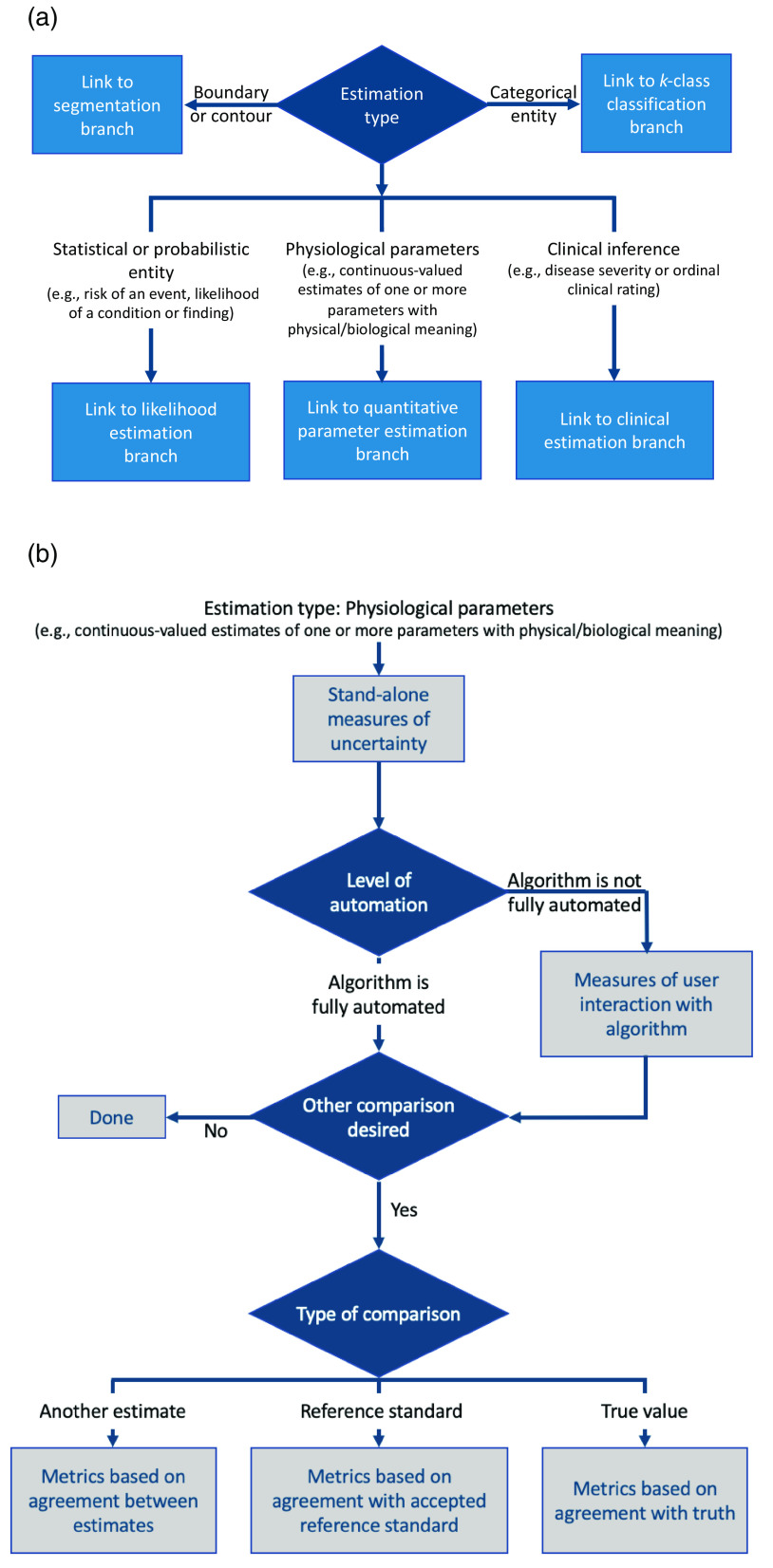
(a) Simplified flowchart of the estimation branch of the MIDRC-MetricTree and (b) an expansion of the physiological parameter estimation sub-branch.

The estimation branch of the MIDRC decision tree (Fig. 8) supports a developer who has created an algorithm intended to provide estimates of one or more parameter values, i.e., measurands.^26^ It is recognized that several scenarios may initially be interpreted by users as estimation tasks, but these pertain to segmentation, e.g., the estimation of a boundary or contour, or classification, e.g., the “estimation” of a categorical Lung-Reporting and Data System score or categorical assessments of COVID-19 severity [Fig. 8(a)], and thus, our decision tree takes the user to the branches for those tasks, discussed in Secs. 3.2 and 3.3. Our treatment of estimation tasks categorizes parameter estimation tasks into three main types: (1) The estimation of a statistical or probabilistic quantity. Examples of this type of estimation task include an algorithm that provides a likelihood of malignancy for a lesion or a patient’s probability or risk of some event or outcome. (2) The estimation of an underlying physiological parameter. This type of estimation task assumes the existence of a true underlying value of the parameter being estimated, where that parameter is defined in “object” or “patient” space, that is, at the input to the imaging system. However, it is not necessary for the user to know the true underlying value. An example is a real, continuous, quantitative estimate of one or more parameters with physical or biological meaning such as the estimation of myocardial perfusion from dynamic contrast-enhanced magnetic resonance imaging. (3) The estimation of a clinical assessment. This type of estimation task undertakes to replicate an interpretation typically performed by a clinician, for example, the clinical assessment of disease severity on a continuous scale. Figure 8(a) encompasses these three estimation tasks. Details are illustrated for the task of estimating an underlying physiological parameter [Fig. 8(b)]; the branches for the tasks of estimating a statistical/probabilistic entity and estimating a clinical assessment have similar structures.

Regardless of the type of estimation task considered here, the quantity to be estimated can be a scalar (a single value) or a vector (multiple values). Examples of scalars include organ or tumor size, bone density, vessel thickness, and extent of organ involvement (e.g., the ratio of the volume of infected and normal regions). Examples of vectors include shape (e.g., in terms of major and minor dimensions of an ellipse, or coefficients for a set of shape basis functions), location (a vector in two or more dimensions), or fluid flow (a vector of velocity and direction).

The estimation branch of the decision tree assists the user in clearly defining the measurand, that is, the parameter to be estimated. In the assessment of the performance of an estimation algorithm, its uncertainty and robustness are important aspects. Measures such as variance, repeatability, and reproducibility serve as indicators of these qualities. They help dissect the spread of estimates to reveal fluctuations originating from diverse sources, such as imaging system noise, imaging sites, devices, operators, protocols, biological variability, and patient positioning, along with their correlations. While the AI algorithm that performs the estimation task may be frequentist or Bayesian, the performance measures described here are frequentist, as is typical for the field.

For the case where there is knowledge of the true or accepted value of the measurand, figures of merit that quantify the accuracy of the estimate can be determined. In particular, the bias in the estimate is the average difference between the mean estimate and the true value.

When the true value of the parameter is unknown, the bias cannot be directly assessed, but agreement (or disagreement) when compared with some reference standard can be used to characterize the estimation algorithm. The comparator, in this case, could be the value of the measurand obtained from a different imaging modality, a different (accepted) estimation algorithm applied to the same imaging data, or a determination by an expert panel’s evaluation of the data. In some circumstances, maximum-likelihood methods can be used to estimate the true value from multiple acquisitions on multiple modalities.

## 4 Discussion and Conclusions

With the advent of AI, several guidelines have been published^27^^–^^30^ or are in development^31^ to help researchers and developers offer relevant information to facilitate the proper evaluation of their work in medical applications. As emphasized in some of these guidelines,^30^ the reporting of performance metrics plays a crucial role that distinguishes a high-quality study from others. The Food and Drug Administration (FDA) has also provided guidance for industry and FDA staff in selected areas, such as in reporting results from studies evaluating diagnostics tests^32^ or in the evaluation of computer-aided detection devices applied to radiology^33^ that include details about metrics for evaluation. Within this larger context of AI/ML evaluation, our work is focused on assisting researchers by providing a user-friendly decision tree and resource identification tool for task-based performance evaluation approaches and metrics.

Other groups and initiatives have been working on guidelines for researchers in this focused area of metric selection and interpretation. In the last part of a series of papers on bias in radiology AI/ML, Faghani et al.^34^ discussed commonly used performance metrics for various tasks in medical imaging. Park et al.^35^ discussed various metrics and graphical methods for evaluating AI/ML performance in radiological diagnosis, as well as essential methodological points to note in using them. Although these studies are excellent tutorials, they are different from our work in that they did not interactively guide the user toward appropriate metrics. In addition, due to their structure, they did not go into detail about several special but important tasks, such as multiclass classification, multilabel segmentation, or estimation. Perhaps closest to our work is Maier-Hein et al.,^36^ who identified inappropriate choice of the problem category (task), poor metric selection, and poor metric application as three core categories related to pitfalls in metric selection. The authors aimed at generating a structured representation of the given problem that captures all properties relevant for metric selection and provided a web-based tool,^37^ currently available as a beta version with restricted access. Their work currently supports problems that can be assigned to one of the following four problem categories: image-level classification, object detection, semantic segmentation, and instance segmentation. A major difference between our work and Maier-Hein et al.^36^ is that we consider other tasks, such as TTE and estimation in our decision tree. It is expected that our work and similar efforts in the literature will interact and provide synergy toward a more comprehensive coverage of medical imaging tasks.

This paper presented a high-level overview of the MIDRC-MetricTree.^2^ Many more details are available in the decision tree itself. It is important to note that, while the MIDRC-MetricTree serves as a resource for selecting appropriate performance metrics for medical image analysis AI/ML, there are several other crucial aspects that were out of scope in this work. First, we developed the MIDRC-MetricTree for medical image analysis tasks and, so far, have excluded other medical imaging tasks such as image reconstruction, image fusion, image registration, or dosimetry, although some of the included metrics are relevant to those areas as well. Moreover, we did not consider medical imaging modalities with a video aspect such as fluoroscopy or endoscopy. The MIDRC-MetricTree does not address aspects such as interpretability/explainability or bias, which are important in developing high-performing, generalizable,^5^ and equitable ^38^ AI/ML models. Also, the MIDRC-MetricTree is not intended to address the comparison of performance among different algorithms using statistical tests (such as done by Obuchowski et al.^39^) or intended to provide guidance on proper AI/ML study design. In addition, there are various other criteria to consider when evaluating AI/ML models in medical imaging, such as device usability, integration into the clinical environment, run time, interpretability, and overall clinical impact on patient outcomes. Our work discusses only the performance assessment of stand-alone AI/ML and not its interaction with, or impact on, human readers (radiologists), which are very important topics of their own in AI/ML evaluation. For example, for algorithms intended to improve a human reader’s performance in estimation tasks, useful figures of merit include reduced bias and improved precision with respect to the manual (unaided reader) performance. For algorithms that either require or allow for manual interaction, it is important to evaluate the algorithm’s performance in the hands of multiple users to determine average performance and variability across readers in terms of inter- and intrareader variability. Useful companion figures of merit for semi-automated algorithms include the fraction of cases where the user chose to correct the algorithm (a measure of the quality of the algorithm) and the time needed for manual entries and adjustments to the algorithm prior to accepting its final output (a human-factor assessment of the usability of the algorithm).

Our work also had certain within-scope limitations. One limitation pertained to effectively handling high variability or unreliability in a reference standard, which often occurs in classification or segmentation tasks involving multiple human experts. While involving multiple experts is desirable, it can introduce challenges in selecting appropriate metrics due to the resulting variability. It should also be noted that the level of variability/reliability of a reference standard may not be known, for example, when downloading a public dataset with one annotated “truth.” Another limitation was that certain aspects of the MIDRC-MetricTree are still areas of active research, and the decision tree itself remains a work in progress. Examples include performance evaluation of multiclass classification tasks with ordinal outputs and estimation of statistical or probabilistic quantities. While we have proposed approaches to address these gaps, it is important to acknowledge that there are no widely accepted “best” metrics for these situations, and the limited literature contributed to the lack of definitive recommendations. Further research and exploration are needed to develop more robust metrics and approaches to overcome these limitations.

In summary, we have developed the MIDRC-MetricTree,^2^ an interactive decision tree metrology tool that is publicly available to aid researchers in conducting task-specific performance evaluations of medical imaging AI/ML algorithms. This decision tree focuses on metrics for measuring AI/ML model performance in five clinical tasks (classification, detection/localization, segmentation, TTE analysis, and estimation). Exploring criteria and metrics in other dimensions of evaluation could complement our work. We encourage interested individuals to visit the MIDRC-MetricTree^2^ for more details, including a comprehensive list of references, software and/or code recommendations, and tutorial videos. Feedback on the decision tree is welcome and collected on the webpage.

## Data Availability

There are no data associated with this paper. The MIDRC-MetricTree^2^ tool is available to all for free.
